# Fatty liver disease determines the progression of coronary artery calcification in a metabolically healthy obese population

**DOI:** 10.1371/journal.pone.0175762

**Published:** 2017-04-18

**Authors:** Yu Mi Kang, Chang Hee Jung, Yun Kyung Cho, Seung Eun Lee, Min Jung Lee, Jenie Yoonoo Hwang, Eun Hee Kim, Joong-Yeol Park, Woo Je Lee, Hong-Kyu Kim

**Affiliations:** 1 Department of Internal Medicine, Asan Medical Center, University of Ulsan College of Medicine, Seoul, Republic of Korea; 2 Department of Health Screening and Promotion Center, Asan Medical Center, University of Ulsan College of Medicine, Seoul, Republic of Korea; University of Colorado Denver School of Medicine, UNITED STATES

## Abstract

**Objectives:**

Metabolically healthy obese (MHO) phenotype describes an obese state with a favorable metabolic profile. However, the prognosis of this subpopulation remains controversial. We aimed to examine whether MHO phenotype is associated with progression of atherosclerotic activity, reflected as the changes in coronary artery calcification (CAC) over time. If so, we sought to determine the role of fatty liver disease (FLD), the hallmark of hepatic steatosis, in this progression.

**Methods:**

We enrolled 1,240 asymptomatic subjects who underwent repeated CAC score measurement during routine health examinations. CAC score progression was defined as either incident CAC in a population free of CAC at baseline, or an increase by ≥2.5 units between the baseline and final square root of CAC scores in participants with detectable CAC at baseline. Subjects were stratified by body mass index (cut-off, 25.0 kg/m^2^) and metabolic health state using Adult Treatment Panel-III criteria. FLD was assessed via ultrasonography.

**Results:**

Over 2.9 years of follow-up, 25.2% of total subjects exhibited CAC score progression. The MHO phenotype was not significantly associated with CAC score progression (multivariate adjusted-odds ratio [OR], 1.45; 95% confidence interval [CI], 0.93–2.25), as compared to the metabolically healthy non-obese (MHNO) phenotype. However, subgroup analysis indicated that the MHO/FLD phenotype was significantly associated with CAC score progression (multivariate adjusted-OR, 2.37; 95% CI, 1.34–4.16), as compared to the MHNO/no FLD phenotype, whereas the MHO/no FLD phenotype was not (multivariate adjusted OR, 1.25; 95% CI, 0.71–2.24).

**Conclusions:**

Obese individuals with FLD have an increased risk of atherosclerosis progression, despite their healthy metabolic profile. Preventive interventions targeting cardiometabolic risk factors should be considered in such individuals, regardless of the weight status.

## Introduction

Obesity is a major health burden worldwide, and is closely associated with an increased risk of developing cardiometabolic co-morbidities, such as type 2 diabetes and cardiovascular diseases (CVDs), which consequently lead to increased mortality [1]. Certain studies have attempted to distinguish an obesity phenotype termed as “metabolically healthy obese (MHO)” state, which is considered relatively benign in nature, given that some individuals within this category display a relatively favorable cardiometabolic profile and similar risk of cardiovascular morbidity and mortality, as compared to normal weight individuals [2].

However, longitudinal studies investigating the long-term prognosis, including cardiovascular outcomes, of the MHO population have shown inconsistent results [3–6]. For example, earlier epidemiological studies have reported an unincreased risk of developing CVD in MHO individuals, as compared to metabolically healthy non-obese (MHNO) individuals [3], whereas a meta-analysis of eight longitudinal studies showed an increased risk of all-cause and cardiovascular mortality in MHO individuals, when compared to MHNO individuals [6]. In addition, clinical studies involving imaging results between these populations also demonstrated controversial findings [4, 5]. For instance, a study reported a significantly larger subclinical CVD burden, as measured by common carotid artery intima-media thickness, in middle-aged MHO women [5], whereas a study of 4,009 asymptomatic subjects showed that the degree of subclinical coronary atherosclerosis, detected via coronary multidetector computed tomography (MDCT), is significantly increased in MHO individuals, as compared to MHNO individuals [4]. However, the cross-sectional nature of the aforementioned reports involving imaging studies may limit the accurate interpretation of the long-term prognosis of the MHO phenotype.

Fatty liver disease (FLD) is closely associated with obesity [7], and has been emphasized as an important cardiovascular risk factor, considering its association with an increased incidence of atherosclerotic changes and coronary heart disease [8]. Similarly, a lower level of liver lipids—a marker of pathological ectopic fat accumulation [9]—has been suggested as one of the most important mechanisms underlying the healthy cardiometabolic profile in MHO individuals [10]. In a longitudinal study of MHO individuals, the presence of FLD increased the risk of developing type 2 diabetes, as compared to that in individuals without FLD, despite the presence of a healthy metabolic profile, by definition [9, 11]. However, thus far, there has been a lack of longitudinal studies that evaluate the progression of atherosclerosis in MHO individuals, as well as the role of FLD in this progression.

Therefore, in the present study, we aimed to assess whether the MHO phenotype is associated with the progression of subclinical atherosclerotic activity, reflected as the dynamic changes in coronary artery calcification (CAC) over time. Furthermore, we sought to determine the role of FLD—the hallmark of hepatic steatosis—in this progression.

## Methods

### Study population

In this study, we enrolled 7,300 individuals who underwent both coronary computed tomography angiography (CCTA) using a 64‐slice MDCT scanner and hepatic ultrasound during routine health evaluations at Asan Medical Center (Seoul, Republic of Korea) between January 2007 and June 2011. Among these individuals, repeated CCTA was performed in 1,591 cases through December 2014. Data was acquired at baseline and also during in‐person follow‐up examinations, as described elsewhere [12]. Questionnaires were completed by every subject, describing the previous medical and/or surgical diseases, medications, and drinking and smoking habits. Drinking habits were categorized as frequency per week (i.e., ≤ once/week and ≥ twice/week [moderate drinker]), smoking habits were categorized as noncurrent or current, and exercise habits were categorized as frequency per week (i.e., ≤ twice/week and ≥ 3 times/week [physically active]), as described previously [4].

The history of CVD was based on each participant's description of their prior events associated with physician‐diagnosed angina, myocardial infarction, and/or cerebrovascular accidents. Subjects were considered having type 2 diabetes if a fasting plasma glucose (FPG) level was ≥7.0 mmol/L and/or a hemoglobin A1c (HbA1c) level ≥6.5% [13], or if they reported the use of antidiabetic medications on the questionnaire [14]. Hypertension was defined as systolic and/or diastolic blood pressure (BP) ≥140/90 mm Hg or the use of hypertensive medications. The 10-year Framingham risk scores (FRS) [15] and atherosclerotic cardiovascular disease (ASCVD) risk scores [16] were calculated as previously described.

Participants with a history of CVD at baseline examinations (n = 95), and those receiving statins (n = 238) were excluded. Also, those who underwent procedures such as percutaneous coronary intervention (n = 8) and coronary arterial bypass surgery (n = 3) after the initial examinations were also excluded. Subjects who were not aged between 20 and 79 years (n = 3), and those with liver cirrhosis or hepatocellular carcinoma (n = 4) or with a transplanted liver (n = 2) were finally excluded. Since some participants met more than 2 exclusion criteria, a total of 1,240 subjects with a mean age of 54.2 years (range, 33–79 years) were finally enrolled in the analysis. All participants provided written informed consent, and this study was approved by the institutional review board of Asan Medical Center.

### Clinical and laboratory measurements

Detailed information regarding the clinical and laboratory measurements is available in the supplemental methods (S1 File).

### Definitions of metabolic health and obesity states

Obesity was defined according to Asia-Pacific body mass index (BMI) cut-off (≥25 kg/m^2^) that were established by the World Health Organization Western Pacific Region [17], and officially adopted by the Korean Centers for Disease Control and Prevention and other Korean government organizations [18]. For the present analyses, metabolically healthy individuals were identified based on the Adult Treatment Panel- III (ATP-III) definition of metabolic syndrome, as having <2 of the following risk factors [19]: (1) a systolic BP ≥ 130 mmHg and/or a diastolic BP ≥ 85 mmHg, or receiving antihypertensive treatment; (2) triglycerides (TG) ≥ 1.7 mmol/L; (3) FPG ≥ 5.6 mmol/L (impaired fasting glucose, IFG) and/or taking antidiabetic medications; and (4) high-density lipoprotein-cholesterol (HDL-C) <1.0 mmol/L in men and <1.3 mmol/L in women. The waist circumference (WC) criterion was not included due to its collinearity with BMI [20]. According to these criteria, study participants were categorized into 1 of 4 groups: (1) MHNO, BMI <25 kg/m^2^ and <2 metabolic risk factors; (2) metabolically unhealthy, non-obese (MUNO), BMI <25 kg/m^2^ and ≥2 metabolic risk factors; (3) MHO, BMI ≥25 kg/m^2^ and <2 metabolic risk factors; or (4) metabolically unhealthy obese (MUO), BMI ≥25 kg/m^2^ and ≥2 metabolic risk factors.

### Diagnosis of FLD

At baseline, subjects underwent hepatic ultrasonography (Ultrasound Systems IU22, Philips, Netherlands). Experienced radiologists performed hepatic ultrasonography, being blinded to the laboratory and clinical details of the study participants. Fatty liver was diagnosed based on the characteristic ultrasonographic features consistent with “bright liver” and an evident contrast between hepatic and renal parenchyma, vessel blurring, focal sparing, and narrowing of the lumen of the hepatic veins [21].

### Use of MDCT for CAC score assessment

At baseline, participants underwent MDCT examinations by either 64‐slice, single‐source, computed tomography (CT; LightSpeed VCT; GE, Milwaukee, WI) or dual‐source CT (Somatom Definition or Somatom Definition Flash; Siemens, Erlangen, Germany), as previously described [4, 12]. An automated software program with the Agatston scoring method [22] was used to calculate the CAC score. The cut‐off points previously described by Greenland et al [23] (i.e., none, 0; mild, 1–100; moderate, and 101–300; severe, >300) were adopted.

### Estimating changes in the CAC score

The progression of CAC was defined differently according to subjects’ baseline MDCT findings. First, if the subjects did not have detectable CAC at baseline, development of incident CAC (i.e., a baseline Agatston score of zero that converted to detectable CAC) at the follow‐up examination was classified as the CAC progressors [24, 25]. In subjects who already had detectable CAC at baseline, the CAC progression was defined as an increase by ≥2.5 units between the baseline and final square root of the CAC scores, as suggested by Hokanson et al. [26–28]. A change of <2.5 units between the baseline and final square root of the CAC score was considered to be within the margin of error for the estimation of the CAC score using MDCT, and was thus attributed to interscan variability; such participants were classified as non-progressors [26–28].

### Statistical analysis

Continuous variables with normal and skewed distributions are expressed as the mean ± standard deviation, and as the median (interquartile range), respectively, whereas categorical variables are expressed as proportions (%). Comparison of the demographic and biochemical characteristics of the study population according to the metabolic health and obesity states was made using one-way analysis of variance (ANOVA) with Scheffe’s method as post hoc analysis, or the Kruskal–Wallis test with the Dunn procedure for continuous variables, and the chi-square test for categorical variables as post hoc analysis.

In order to calculate the adjusted odds ratios (ORs) for CAC score progression according to the metabolic health and obesity states, a logistic regression model was applied. The results of the analyses are presented as ORs with 95% confidence intervals (CIs), compared with the MHNO group as the reference. All statistical analyses were performed using SPSS version 18.0 for Windows (SPSS Inc., Chicago, IL). A *p* value of <0.05 was considered statistically significant.

## Results

### Baseline characteristics of the study subjects

The baseline clinical and biochemical characteristics of the included subjects according to the metabolic health defined by ATP-III criteria and BMI are summarized in Table 1. Among the included subjects, 22.7% exhibited the MHO phenotype. The mean age of all subjects was 54.2 ± 7.4 years, and that among each metabolic health-BMI category did not differ significantly.

**Table 1:** Baseline clinical and biochemical characteristics of the study subjects according to metabolic health defined by the ATP-III criteria and obesity.

	Non-obese		Obese		
	Metabolically healthy (MHNO)	Metabolically unhealthy (MUNO)	Metabolically healthy (MHO)	Metabolically unhealthy (MUO)	
Variables	(n = 447)	(n = 195)	(n = 282)	(n = 316)	*p* for trend
**Age (years)**	54.1 ± 7.5	54.6 ± 7.4	54.2 ± 7.7	53.9 ± 7.0	0.700
**Sex (male, %)**	70.9	81.5^a^	86.2^a^	93.0	<0.001
**BMI (kg/m**^**2**^**)**	22.7 ± 1.8	23.3 ± 1.4	26.9 ± 1.7	27.5 ± 2.8	<0.001
**WC (cm)**	81.1 ± 6.3	83.9 ± 5.5	91.8 ± 5.5^a^	93.0 ± 6.8^a^	<0.001
**SBP (mmHg)**	114.1 ± 11.5	124.2 ± 12.2^a^	118.0 ± 10.9	125.9 ± 12.8^a^	<0.001
**DBP (mmHg)**	72.5 ± 9.6	80.6 ± 10.3^a^	75.3 ± 9.0	81.6 ± 10.5^a^	<0.001
**Current smoker (%)**	23.5^a^^b^	29.7^a^^c^^b^	24.8^b^^c^	32.9^d^	0.021
**Moderate drinker (%)**	42.1	54.9^a^	53.5^a^	66.5	<0.001
**Physically active (%)**	44.8	47.1	45.4	38.0	0.209
**FPG (mmol/L)**	5.4 ± 0.6	6.4 ± 1.5^a^	5.6 ± 0.7	6.5 ± 1.1^a^	<0.001
**HbA1c (%)**	5.4 (5.2–5.7)^a^	5.7 (5.4–6.2)^b^	5.5 (5.2–5.7)^a^	5.7 (5.4–6.1)^b^	<0.001
**Diabetes (%)**	4.5^a^	27.7^b^	7.8^a^	22.2^b^	<0.001
**Total cholesterol (mmol/L)**	5.2 ± 0.8	5.2 ± 0.9	5.1 ± 0.8	5.1 ± 0.9	0.547
**TG (mmol/L)**	1.0 (0.8–1.3)	1.8 (1.1–2.2)	1.2 (1.0–1.5)	1.9 (1.4–2.5)	<0.001
**LDL-C (mmol/L)**	3.2 ± 0.7	3.3 ± 0.8	3.3 ± 0.7	3.3 ± 0.8	0.921
**HDL-C (mmol/L)**	1.5 ± 0.3	1.3 ± 0.3	1.4 ± 0.3	1.2 ± 0.3	<0.001
**Uric acid (μmol/L)**	5.3 ± 1.3	5.7 ± 1.4^a^	6.0 ± 1.3^a^	6.3 ± 1.3	<0.001
**AST (U/L)**	24.0 (21.0–29.0)	25.0 (22.0–32.0)^a^	26.0 (22.0–32.0)^a^	27.0 (23.0–33.0)	<0.001
**ALT (U/L)**	20.0 (15.0–25.0)	22.0 (18.0–32.0)^a^	24.0 (18.0–32.0)^a^	28.0 (21.0–37.0)	<0.001
**GGT (U/L)**	18.0 (13.0–28.0)	28.0 (19.0–45.0)^a^	25.5 (17.0–42.0)^a^	34.0 (24.0–49.0)	<0.001
**hsCRP (mg/L)**	0.5 (0.3–0.9)	0.7 (0.4–1.6)^a^^b^	0.7 (0.4–1.4)^a^	0.8 (0.4–1.5)^b^	<0.001
**HOMA-IR**	1.3 (0.9–1.8)	1.8(1.2–2.6)^a^	1.8 (1.2–2.7)^a^	2.5 (1.9–4.0)	<0.001
**10-year FRS (%)**	5.0 (1.0–8.0)	8.0 (4.0–12.0)	6.0 (4.0–10.0)	10.0 (6.0–12.0)	<0.001
**10-year ASCVD (%)**	3.8 (1.7–7.1)	7.0 (3.3–12.6)^a^	5.1 (2.6–8.4)	8.0 (4.9–12.2)^a^	<0.001
**Baseline CAC score**	0.00 (0.00–7.00)	0.00 (0.00–23.00)	0.00 (0.00–18.55)	2.00 (0.00–61.00)	<0.001
**Baseline CAC score category**					
**0 (n, %)**	304 (68.0)	100 (51.3)	181 (57.1)	147 (46.5)	
**1–100 (n, %)**	101 (22.6)	71 (36.4)	97 (34.4)	113 (35.8)	
**101–300 (n, %)**	20 (4.5)	18 (9.2)	16 (5.7)	38 (12.0)	
**>300 (n, %)**	22 (4.9)	6 (3.1)	8 (2.8)	18 (5.7)	
**Presence of FLD (n, %)**	105 (23.5)	90 (46.2)^a^	142 (50.4)^a^	234 (74.1)	<0.001
**Follow-up interval (years)**	3.0 (2.1–3.9)	2.8 (2.0–3.3)	3.0 (2.1–3.9)	2.9 (2.0–3.7)	0.059

Data are presented as n (%), median (interquartile range), or mean±SD. BMI indicates body mass index; WC, waist circumference; SBP, systolic blood pressure; DBP, diastolic blood pressure; FPG, fasting plasma glucose; TG, triglycerides; LDL-C, LDL-cholesterol; HDL-C, HDL-cholesterol; AST, aspartate aminotransferase; ALT, Alanine aminotransferase; GGT, Gamma-glutamyltransferase; hsCRP, high-sensitivity C-reactive protein; HOMA-IR, homeostatic model assessment of insulin resistance; FRS, Framingham risk score; ASCVD, atherosclerotic cardiovascular disease; CACS, coronary artery calcification score and FLD, fatty liver disease.

^a,^ The same letters indicate a statistically insignificant difference.

^b,^ The same letters indicate a statistically insignificant difference.

^c,^ The same letters indicate a statistically insignificant difference.

^d^The same letters indicate a statistically insignificant difference.

Compared with the MHNO group, a greater proportion of male subjects was noted in the MHO group, and these individuals exhibited a less favorable risk profile, such as higher levels of FPG, TG, and low-density lipoprotein-cholesterol (LDL-C) and lower levels of HDL-C and homeostatic model assessment of insulin resistance (HOMA-IR) (Table 1). There was no significant difference in the self-reported lifestyle habits (i.e., drinking, smoking, and the level of physical activity) between individuals of the MHO and MHNO groups. Compared with the MUNO group, the MHO group displayed a more favorable risk profile, including lower TG, FPG, and HbA1c, and higher HDL-C levels, despite their higher BMI and WC (Table 1).

The mean aspartate aminotransferase (AST), alanine aminotransferase (ALT), and gamma-glutamyltransferase (GGT) levels at baseline were within the normal ranges, and did not significantly differ between the MUNO and MHO groups. The 10-year FRS and ASVCD risk scores were highest in MUO individuals, and decreased in the following order: MUNO, MHO, and MHNO (Table 1).FLD was more prevalent in MUO individuals (74.1%), whereas the prevalence of FLD was similar between MUNO and MHO individuals. The MUO group showed a significantly higher baseline CAC score (Table 1).

### Association of metabolic health and obesity status with CAC progression

**Fig 1:**
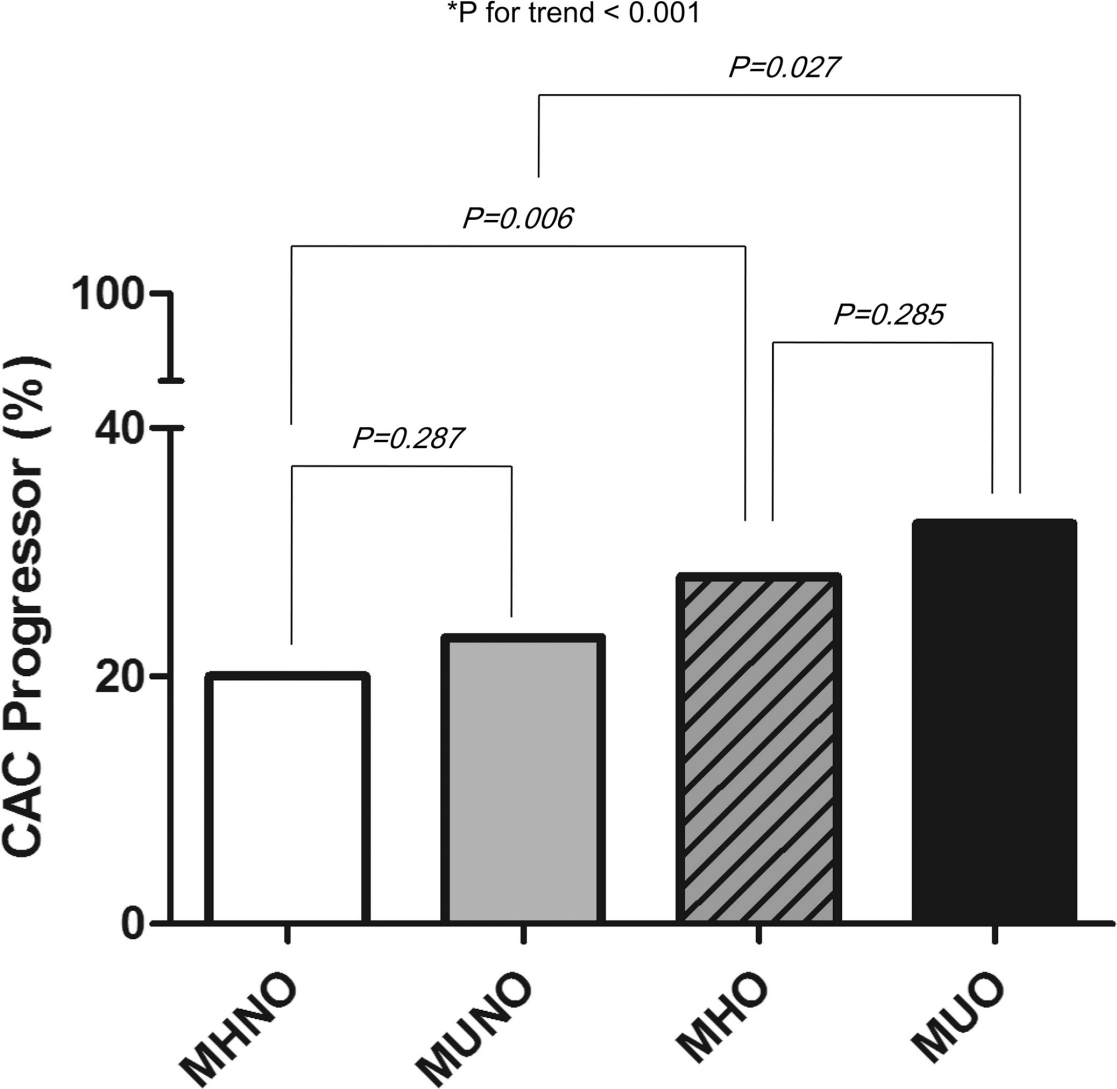
Percentage of coronary artery calcification progressors in the 4 categories of metabolic health and obesity.

During the median follow-up of 2.9 years (range, 0.8–7.0 years), 312 of 1,240 subjects (25.2%) exhibited CAC progression. As expected, the proportion of CAC progressors was the highest (32.3%, 120/316) in the MUO group, and gradually decreased in the following order: MHO, MUNO, and MHNO groups (28.0% [79/282], 23.1% [45/195], and 19.2% [86/447], respectively; Fig 1).

To assess the degree of the association between each metabolic health and obesity phenotype with CAC progression, we analyzed the ORs of CAC progression in each metabolic health-BMI category (Table 2). Compared with MHNO individuals, MUNO individuals did not show a significantly higher association with CAC progression, whereas the ORs for CAC progression in the MUO group remained significantly high even after adjusting for possible confounders (multivariate-adjusted OR, 1.71 [95% CI, 1.10–2.65]) (Table 2). In contrast, the MHO individuals had a significantly higher OR for CAC progression (unadjusted OR, 1.63 [95% CI, 1.15–2.32]); however, this statistical significance disappeared after multivariate analysis (multivariate-adjusted OR, 1.45 [95% CI, 0.93–2.25]; Table 2).

**Table 2:** Odds ratios (ORs) and 95% confidence intervals (CI) for progression of coronary calcification according to the metabolic health and obesity states.

	Non-obese		Obese		*p* for trend
	Metabolically healthy (MHNO)	Metabolically unhealthy (MUNO)	Metabolically healthy (MHO)	Metabolically unhealthy (MUO)	
Subgroup	(n = 447)	(n = 195)	(n = 282)	(n = 316)	
ORs for CAC progression					
Unadjusted	1	1.26 (0.84−1.89)	1.63 (1.15−2.32)	2.00 (1.43−2.32)	<0.001
Model 1	1	1.14 (0.74−1.73)	1.40 (0.92−2.14)	1.66 (1.09−2.53)	<0.001
Model 2	1	1.11 (0.73−1.71)	1.42 (0.93−2.18)	1.62 (1.06−2.49)	<0.001
Model 3	1	1.19 (0.76−1.84)	1.45 (0.93−2.25)	1.71 (1.10−2.65)	<0.001

Data are expressed as OR (95% confidence interval). CAC indicates coronary artery calcification.

Model 1: adjusted for age, sex, and waist circumference.

Model 2: adjusted for variables in Model 1 as well as drinking, smoking, and exercise habits.

Model 3: adjusted for variables in Model 2 as well as baseline CAC score, LDL-C, hsCRP, and follow-up interval.

### Influence of baseline FLD on the association of metabolic health and obesity status with CAC progression

We assessed the combined effect of the metabolic health and obesity state, as well as the presence of FLD on the progression of atherosclerosis. MHNO individuals without baseline FLD had the lowest incident rate of CAC progression (2.2%). CAC progressed only in 2.5% (31/140) of MHO individuals without baseline FLD, whereas MHO individuals with FLD at baseline showed an elevated incidence rate of CAC progression (3.9%, 48/142), although this value was still lower than that of MUO individuals with baseline FLD (5.9%, 73/234).

**Fig 2:**
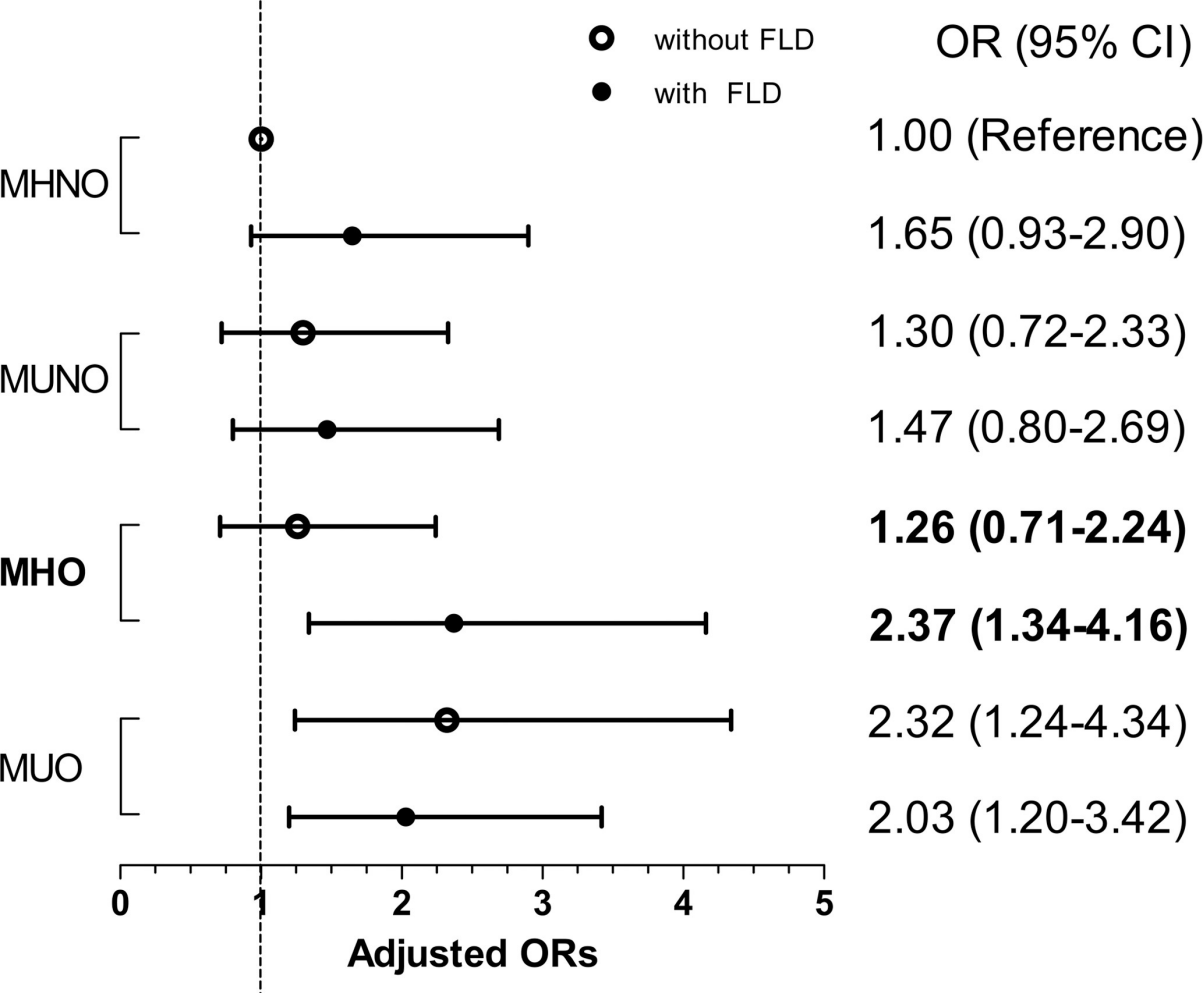
Illustration of the odds ratios (ORs) and 95% confidence intervals (CI) for the progression of coronary artery calcification according to metabolic health, obesity, and the presence of fatty liver disease. The ORs were adjusted for age, sex, waist circumference, drinking, smoking, exercise habits, baseline CAC score, LDL-C, hsCRP, and follow-up interval.

Table 3 shows the ORs for CAC progression according to the baseline metabolic health and obesity state, as well as the presence of FLD. Even after adjusting for multiple, potentially-confounding variables, MUO individuals showed significantly higher ORs (adjusted ORs, 2.32 [95% CI, 1.24–4.34] in the MUO-no FLD group, and 2.03 [1.20–3.42] in the MUO-FLD group), irrespective of the presence of baseline FLD (Table 3 and Fig 2). Interestingly, a statistically significant association was selectively observed in the MHO individuals with baseline FLD (multivariate-adjusted OR, 2.37 [95% CI, 1.34–4.16]), but not in those without baseline FLD (multivariate-adjusted, OR 1.26 [95% CI, 0.71–2.24]; Table 3 and Fig 2).

**Table 3:** Odds ratios (ORs) and 95% confidence intervals (CI) for progression of coronary calcification according to metabolic health, obesity, and the presence of fatty liver disease.

	Non-obese				Obese				*p* for trend
	Metabolically healthy (MHNO)		Metabolically unhealthy (MUNO)		Metabolically healthy (MHO)		Metabolically unhealthy (MUO)		
	(n = 447)		(n = 195)		(n = 282)		(n = 316)		
Subgroup	No FLD (n = 342)	FLD (n = 105)	No FLD (n = 105)	FLD (n = 90)	No FLD (n = 140)	FLD (n = 142)	No FLD (n = 82)	FLD (n = 234)	
ORs for CAC progression									
Unadjusted	1	2.04 (1.23−3.41)	1.35 (0.78−2.35)	1.75 (1.01−3.05)	1.45 (0.89−2.37)	2.61 (1.66−4.09)	2.79 (1.64−4.78)	2.32 (1.56−3.45)	<0.001
Model 1	1	1.65 (0.96−2.83)	1.25 (0.71−2.20)	1.42 (0.80−2.54)	1.24 (0.71−2.15)	2.29 (1.33−3.95)	2.23 (1.22−4.08)	2.01 (1.21−3.33)	<0.001
Model 2	1	1.69 (0.98−2.91)	1.21 (0.69−2.15)	1.42 (0.79−2.54)	1.25 (0.71−2.18)	2.38 (1.37−4.12)	2.22 (1.21−4.09)	1.96 (1.18−3.27)	<0.001
Model 3	1	1.65 (0.93−2.90)	1.30 (0.72−2.33)	1.47 (0.80−2.69)	1.26 (0.71−2.24)	2.37 (1.34−4.16)	2.32 (1.24−4.34)	2.03 (1.20−3.42)	<0.001

Data are expressed as OR (95% CI). CAC indicates coronary artery calcification.

Model 1: adjusted for age, sex, and waist circumference.

Model 2: adjusted for variables in Model 1 as well as drinking, smoking, and exercise habits.

Model 3: adjusted for variables in Model 2 as well as baseline CAC score, LDL-C, hsCRP, and follow-up interval.

## Discussion

In our present study of a large number of asymptomatic subjects, 22.7% subjects displayed the MHO phenotype, as defined by the ATP-III criteria and BMI. Over a median follow-up duration of 2.9 years, approximately a quarter of all the subjects and 28.0% of MHO subjects exhibited CAC progression (Fig 1). The MHO state did not exhibit a significant association with CAC progression after multivariate analysis, as compared to the MHNO state (Table 2). However, when MHO individuals were further divided according to the presence of FLD at baseline, those with FLD showed a significant association with CAC progression (multivariate-adjusted OR, 2.37 [95% CI, 1.34–4.16]), whereas those without FLD did not (multivariate adjusted OR, 1.26 [95% CI, 0.71–2.24]; Table 3 and Fig 2). These results support our hypothesis that the presence of FLD could serve as a risk determinant in the progression of subclinical atherosclerosis in MHO individuals.

Over the past few decades, studies have focused on the question of whether all obesity is equal in nature; however, conflicting evidence has been obtained regarding the long-term course of the MHO population [3–6]. In contrast to earlier epidemiological studies that showed either unincreased or slightly increased risks of CVD for the MHO phenotype [3, 29–31], more recent studies have indicated significantly worse cardiometabolic outcomes in the MHO population [6, 32, 33]. Furthermore, studies have consistently reported that a certain proportion of MHO individuals develop incident hypertension [20], type 2 diabetes [34], and chronic kidney diseases [35] to a significant extent, as compared to the MHNO population. This heterogeneity in the outcomes of the MHO population suggests that not all MHO individuals follow the same metabolic course, and raised concerns regarding the precision of the current definition of metabolic health in predicting individuals with worse cardiometabolic outcomes [20].

The fact that certain obese individuals with healthy metabolic profiles are at a greater risk of adverse cardiovascular outcomes indicates the presence of an undiscovered risk determinant of worse cardiometabolic outcomes. Thus far, features such as less visceral adiposity, less systemic inflammation, and increased aerobic fitness have been proposed as the mechanisms underlying preserved metabolic health in some obese individuals [36], and among these, the amount of liver lipids was found to be the most important determinant of insulin sensitivity in obese individuals [10]. Moreover, patients with non-alcoholic FLD (NAFLD) have a higher prevalence of atherosclerosis, as compared to those without NAFLD, independent of obesity and other established risk factors [37], thus highlighting the role of NAFLD as a potential independent CVD risk factor. Although the association of FLD with poor cardiovascular outcomes is well established [38], no study has observed CAC progression within the MHO population over time thus far. Interestingly, our results did not reveal a significant association between the MHO phenotype and subclinical atherosclerosis progression until we used FLD as an additional risk determinant (Table 3 and Fig 2). Therefore, our current study results suggest that, despite the healthy metabolic profile, the presence of FLD can potentially lead to atherosclerotic diseases in an obese population, and that FLD should be considered as one of the major criteria in the definition of metabolic health.

Although the exact mechanism through which FLD leads to CAC progression was not evaluated in the present study, the development and progression of insulin resistance as supported by higher median HOMA-IR in subjects with FLD than that in subjects without FLD at baseline (2.21 vs. 1.40; S1 Table) might have served as the key mediators [39]. Increased liver fat content, one of the best independent predictors of hepatic and peripheral insulin resistance, can lead to deranged glucose, fatty acid, and lipoprotein metabolism [8]. Therefore, the subsequent deleterious effects of high insulin resistance, such as increased oxidative stress and subclinical inflammation, worsened adipokine profile, hypercoagulability, and endothelial dysfunction could have further accelerated the atherosclerotic activity [8].

Despite the controversy related to the prognosis of MHO, it is evident that particular attention should be given to metabolically unhealthy subjects within the non-obese population (i.e., MUNO) [6, 29]. The MUNO population has consistently shown an unfavorable prognosis, regardless of the type of health-related outcomes being studied and the increased mortality [6, 29]. This group may represent the most severe subtype along the phenotypic spectrum of individuals genetically predisposed to CVD, considering that they have unfavorable metabolic features even without having excess weight [6]. However, in our current study, MUNO subjects did not display an increased risk of CAC progression (Tables 2 and 3 and Fig 2); this may be due to the limited number of subjects categorized as MUNO.

The present study had certain limitations of note. First, despite the large cohort size, our subjects did not necessarily represent the general Korean or Asian population due to the voluntary nature of the recruitment. Hence, it is possible that more high‐risk participants would be more likely to repeat their CAC measurement. Second, we used CAC score progression, as detected by MDCT, as a measure to screen for early atherosclerotic activity in the subclinical stages, rather than actual major cardiovascular events. Hence, our results should be supported by a long-term follow up study with definite outcomes to determine whether the CAC progression in MHO individuals with FLD observed in the present study eventually leads to major cardiovascular events and CVD-related mortality. Third, we adopted the SQRT method to determine CAC progression; however, there is no clear consensus on the best definition for CAC progression. Nevertheless, a growing body of evidence suggests that the SQRT method is the best CAC progression model to predict mortality, and that a SQRT difference of 2.5 provides the best fit for the data [26]. Fourth, in our study, the diagnosis of FLD was made by morphologic changes detected by ultrasound, although the gold standard is a liver biopsy. Although it is widely used due to its low cost, safety and accessibility, ultrasonography-based diagnosis of FLD requires equipment and radiologic experts, and its specificity is relatively low because it can detect hepatic steatosis only when it is greater than 25–30% [40]. It is quite inferior to other modalities such as magnetic resonance spectroscopy, which can detect the hepatic steatosis even when hepatic triglyceride level is above 5% [41, 42]. Finally, as we recorded the history of alcohol consumption in a semi-quantitative manner, it was not possible to differentiate alcoholic FLD and NAFLD. However, the relative contribution of alcohol consumption in the development of FLD remains controversial [43].

Despite these limitations, our present study is the first to show the progression of subclinical atherosclerotic activity in a subset of asymptomatic obese individuals with low metabolic risk, and to prove that FLD at baseline can serve as an independent risk determinant for predicting CAC score progression. Thus, an obese population, especially that with FLD, cannot be considered as having a benign condition despite their healthy metabolic profile, and should be followed carefully. Long-term follow-up studies should be conducted to determine whether the CAC progression associated with FLD in MHO state eventually leads to major cardiovascular outcomes and cardiovascular mortality in the future.

## Supporting information

S1 Table Baseline clinical and biochemical characteristics of the study subjects according to metabolic health defined by the ATP-III criteria and obesity. (DOCX)

S1 File Supplemental methods. (DOCX)

## Abbreviations

ALT: alanine aminotransferase
ANOVA: one-way analysis of variance
ASCVD: atherosclerotic cardiovascular disease
AST: aspartate aminotransferase
ATP-III: Adult Treatment Panel-III
BMI: body mass index
BP: blood pressure
CAC: coronary artery calcification
CCTA: coronary computed tomography angiography
CI: confidence intervals
CVD: cardiovascular disease
FLD: fatty liver disease
FPG: fasting plasma glucose
FRS: Framingham risk scores
GGT: gamma-glutamyltransferase
HDL-C: high-density lipoprotein-cholesterol (HDL-C)
HOMA-IR: homeostatic model assessment of insulin resistance
LDL-C: low-density lipoprotein-cholesterol
MDCT: multidetector computed tomography
MHNO: metabolically healthy non-obese
MHO: metabolically healthy obese
MUNO: metabolically unhealthy, non-obese
MUO: metabolically unhealthy obese
NAFLD: non-alcoholic fatty liver disease
OR: odd ratio
TG: triglycerides
WC: waist circumference
