# Supplementary material for: Fatty liver disease determines the progression of coronary artery calcification in a metabolically healthy obese population
Source: PLoS One. 2017 Apr 18;12(4):e0175762. doi: 10.1371/journal.pone.0175762 (PMC5395191; doi:10.1371/journal.pone.0175762)
Supplement: S1 Table — (DOCX) [file pone.0175762.s001.docx]

**Supplemental Table 1.** Baseline clinical and biochemical characteristics of the study subjects according to metabolic health defined by the ATP-III criteria and obesity.

|  | **Total** | **MHO without FLD** | **MHO with FLD** |  |
| --- | --- | --- | --- | --- |
| **Variables** | **(n=1,240)** | **(n=140)** | **(n=142)** | ***p*-value** |
| **Age (years)** | 54.2 ± 7.4 | 55.1 ± 8.1 | 53.4 ± 7.1 | 0.287 |
| **Sex (male, %)** | 81.7 | 80.0 | 92.3 | 0.003 |
| **BMI (kg/m^2^)** | 25.0 ± 3.0 | 26.5 ± 1.4 | 27.2 ± 1.9 | <0.001 |
| **WC (cm)** | 87.0 ± 8.2 | 90.4 ± 4.9 | 93.1 ± 5.8 | 0.039 |
| **Systolic BP (mmHg)** | 119.6 ± 12.8 | 118.2 ± 11.1 | 117.8 ± 10.7 | 0.761 |
| **Diastolic BP (mmHg)** | 76.7 ± 10.5 | 75.2 ± 8.9 | 75.4 ± 9.1 | 0.201 |
| **Current smoker (%)** | 27.2 | 22.1 | 27.5 | 0.336 |
| **Moderate drinker (%)** | 52.9 | 56.4 | 50.7 | 0.342 |
| **Physically active (%)** | 43.5 | 50.0 | 40.8 | 0.151 |
| **FPG (mmol/L)** | 5.8 ± 1.0 | 5.5 ± 0.6 | 5.7 ± 0.8 | 0.002 |
| **HbA1c (%)** | 5.4 (5.1–5.6) | 5.4 (5.1–5.7) | 5.5 (5.3–5.8) | 0.005 |
| **Total cholesterol (mmol/L)** | 5.1 ± 0.8 | 5.1 ± 0.8 | 5.1 ± 0.8 | 0.999 |
| **TG (mmol/L)** | 1.3 (1.0–1.8) | 1.1 (0.9–1.4) | 1.3 (1.0–1.5) | <0.001 |
| **LDL-C (mmol/L)** | 3.3 ± 0.7 | 3.2 ± 0.7 | 3.3 ± 0.7 | 0.453 |
| **HDL-C (mmol/L)** | 1.3 ± 0.3 | 1.4 ± 0.3 | 1.3 ± 0.3 | 0.411 |
| **Uric acid (µmol/L)** | 5.8 ± 1.4 | 5.7 ± 1.3 | 6.2 ± 1.3 | 0.462 |
| **AST (U/L)** | 25 (22–31) | 24 (21–30) | 28 (23–35) | 0.001 |
| **ALT (U/L)** | 23 (17–31) | 21 (16–26) | 28 (20–39) | <0.001 |
| **GGT (U/L)** | 25 (16–40) | 22 (15–38.5) | 28 (20–46.3) | 0.004 |
| **hsCRP (mg/L)** | 0.6 (0.3–1.3) | 0.6 (0.3–1.2) | 0.7 (0.4–1.5) | 0.122 |
| **HOMA-IR** | 1.78 (1.15–2.64) | 1.49 (1.08–2.20) | 2.21 (1.56–2.96) | <0.001 |
| **10-year FRS (%)** | 6.0 (3.0–10.0) | 6.0 (4.0–10.) | 6.0 (4.0–10.) | 0.277 |
| **10-year ASCVD (%)** | 5.5 (2.7–9.7) | 4.8 (2.5–8.2) | 5.5 (2.7–9.0) | 0.337 |
| **Baseline CAC score** | 0.00 (0.00–22.78) | 0.00 (0.00–17.75) | 0.00 (0.00–19.25) | 0.237 |
| **Baseline CAC score (new) >0 (n, %)** | 528 (42.9) | 52 (37.1) | 69 (48.6) | 0.055 |
| **Basline CAC score category** |  |  |  |  |
| **0 (n, %)** | 712 (57.4) | 88 (62.9) | 73 (51.4) |  |
| **1–100 (n, %)** | 382 (30.8) | 40 (28.6) | 57 (40.1) |  |
| **101–300 (n, %)** | 92 (7.4) | 7 (5.0) | 9 (6.3) |  |
| **>300 (n, %)** | 54 (4.4) | 5 (3.6) | 3 (2.1) |  |
| **Follow-up interval (years)** | 3.0 (2.1–3.8) | 3.0 (2.1–3.9) | 3.0 (2.1–4.0) | 0.998 |

Data are presented as n (%), median (interquartile range), or mean ± SD. BMI indicates body mass index; WC, waist circumference; SBP, systolic blood pressure; DBP, diastolic blood pressure; FPG, fasting plasma glucose; TG, triglycerides; LDL-C, LDL-cholesterol; HDL-C, HDL-cholesterol; AST, aspartate aminotransferase; ALT, Alanine aminotransferase; GGT, Gamma-glutamyltransferase; hsCRP, high-sensitivity C-reactive protein; HOMA-IR, homeostatic model assessment of insulin resistance; FRS, Framingham risk score; ASCVD, atherosclerotic cardiovascular disease; MHO, metabolically healthy obesity; and MUO, metabolically unhealthy obesity.
