# Supplementary material for: Fatty liver disease determines the progression of coronary artery calcification in a metabolically healthy obese population
Source: PLoS One. 2017 Apr 18;12(4):e0175762. doi: 10.1371/journal.pone.0175762 (PMC5395191; doi:10.1371/journal.pone.0175762)
Supplement: S1 File — (DOCX) [file pone.0175762.s002.docx]

**Supplemental methods**

**Clinical and laboratory measurements**

Anthropometric examinations were performed while the subjects were wearing light-weight clothing provided by the institution, without any shoes. The body mass index was calculated by dividing the weight in kilograms by the square of the height in meters. The waist circumference was measured midway between the costal margin and the iliac crest at the end of normal expiration. Following a resting period of at least 5 min, blood pressure was measured on the right arm by automatic manometry using a Vital Sign Monitor 300 Series (Welch Allyn Co., Ltd., Beaverton, OR) with an appropriate cuff size. After overnight fasting, blood samples were drawn from subjects’ antecubital veins into vacuum-sealed tubes and were transferred to a central, certified laboratory at Asan Medical Center. Measurements included the concentration of fasting plasma glucose (FPG), insulin, high-sensitivity C-reactive protein (hsCRP), several lipid parameters, and liver enzymes.

Fasting total cholesterol, high-density lipoprotein-cholesterol, low-density lipoprotein-cholesterol, triglycerides, uric acid, aspartate aminotransferase, and alanine aminotransferase were measured by an enzymatic colorimetric method using a Toshiba 200 FR Neo autoanalyzer (Toshiba Medical System Co., Ltd., Tokyo, Japan). Gamma-glutamyltransferase was measured using the L-γ-glutamyl-p-nitroanilide method (Toshiba). HsCRP and FPG were measured using the immunoturbidimetric method (Toshiba) and by an enzymatic colorimetric method using a Toshiba 200 FR autoanalyzer (Toshiba), respectively. Serum insulin was measured by immunoradiometric assay (TFB Co., Ltd., Tokyo, Japan). Ion-exchange high-performance liquid chromatography (Bio-Rad Laboratories, Inc., Hercules, CA) was used to measure HbA1c levels. The intra- and inter-assay coefficients of variation of these analyses were consistently <3.5%. The homeostatic model assessment of insulin resistance was calculated as the product of the fasting serum insulin (μU/mL) and FPG (mmol) concentrations, divided by 22.5. All enzyme activities were measured at 37°C.
